# Impact of DC-Coupled Electrophysiological Recordings for Translational Neuroscience: Case Study of Tracking Neural Dynamics in Rodent Models of Seizures

**DOI:** 10.3389/fncom.2022.900063

**Published:** 2022-07-21

**Authors:** Amirhossein Jafarian, Rob C. Wykes

**Affiliations:** ^1^Department of Clinical Neurosciences and Cambridge University Hospitals NHS Trust, University of Cambridge, Cambridge, United Kingdom; ^2^Department of Clinical and Experimental Epilepsy, UCL Queen Square Institute of Neurology, London, United Kingdom; ^3^Nanomedicine Lab, University of Manchester, Manchester, United Kingdom

**Keywords:** neural mass model, continuous-discrete unscented Kalman filter, DC-coupled electrophysiological recordings, synaptic physiology, infraslow oscillations

## Abstract

We propose that to fully understand biological mechanisms underlying pathological brain activity with transitions (e.g., into and out of seizures), wide-bandwidth electrophysiological recordings are important. We demonstrate the importance of ultraslow potential shifts and infraslow oscillations for reliable tracking of synaptic physiology, within a neural mass model, from brain recordings that undergo pathological phase transitions. We use wide-bandwidth data (direct current (DC) to high-frequency activity), recorded using epidural and penetrating graphene micro-transistor arrays in a rodent model of acute seizures. Using this technological approach, we capture the dynamics of infraslow changes that contribute to seizure initiation (active pre-seizure DC shifts) and progression (passive DC shifts). By employing a continuous–discrete unscented Kalman filter, we track biological mechanisms from full-bandwidth data with and without active pre-seizure DC shifts during paroxysmal transitions. We then apply the same methodological approach for tracking the same parameters after application of high-pass-filtering >0.3Hz to both data sets. This approach reveals that ultraslow potential shifts play a fundamental role in the transition to seizure, and the use of high-pass-filtered data results in the loss of key information in regard to seizure onset and termination dynamics.

## Introduction

This article illustrates the importance of wide-bandwidth electrophysiological recordings, specifically the inclusion of ultraslow potential shifts and infraslow oscillations, for tracking the evolution of synaptic physiology during the paroxysmal transition into and out of seizure. We illustrate that removing (or ignoring) infraslow changes can have a significant effect on inferring neural generators from data, which in turn could influence designing an effective treatment strategy for neurological disorders such as epilepsy. In this study, we neither aim to explore or elucidate true causes of phase transitions from brain activity nor develop new methods to infer physiological parameters. Instead, we provide case examples of reconstructing synaptic parameters from real data to demonstrate the limitations of high-pass-filtered (>0.3 Hz) data sets to infer mechanisms underlying seizure transition.

Infraslow oscillations by definition refer to oscillations in the frequency ranges [0.01, 0.1] *Hz* in electrophysiological recordings. These slow waves were first captured by implanted electrodes in animal studies (Aladjalova, 1957). Biological generators causing these oscillations could be very complex (Watson, 2018). Proposed mechanisms include neuronal sources [e.g., slow after-hyperpolarisation resulting from dynamic changes in Ca^2+^and K^+^ conductance (Kandel and Spencer, 1961; Hotson and Prince, 1980; Jahnsen and Llinás, 1984; Traub et al., 1993)], activity of neurovascular coupling units and glial cells (e.g., induction of long-lasting hyperpolarising potentials due to changes in astrocyte buffering of extracellular K+) (Jefferys, 1995; Yamada et al., 1998; Kuga et al., 2011; Kaiser, 2020), and more widespread network dynamics (Steriade et al., 1993a,b; Drew et al., 2020). The mechanisms underlying infraslow oscillations have been explored in specific brain conditions, for example, sleep rhythms (Achermann et al., 1993; Ruskin et al., 1999; Lemieux et al., 2014; Van Putten et al., 2015), resting states (Damoiseaux et al., 2006; Van Someren et al., 2011), or epilepsy, where fast oscillations may be recruited/modulated by slow activity (De Goede and Van Putten, 2019; Hashimoto et al., 2020, 2021; Bonaccini Calia et al., 2021). In particular, ultraslow potential shifts and slow oscillations may provide valuable clinical information, which may be useful as a biomarker for detecting an epileptogenic zone (Ikeda et al., 2020; Lundstrom et al., 2021).

In addition to experimental studies, computational neuroscientists have established detailed biological, mean field, and phenomenological models to infer mechanistic insights into the underlying generators of infraslow brain signals. Selected examples of biological models include (i) reproducing ultraslow oscillations by short-term synaptic plasticity mechanisms due to the action of dopamine (Kobayashi et al., 2017); (ii) network models of cortical patches based on a model of single neurons for exploring the correlation between slow and fast neuronal activities (Lundqvist et al., 2013); (iii) elucidating the role of Ca^2+^ and K^+^ dynamics in generation of slow waves during resting states (Krishnan et al., 2018); and (IV) exploring the role of neurovascular coupling in production of slow activity in health (Wade et al., 2011; Kozachkov and Michmizos, 2017) or diseased brain states (Tuckwell and Miura, 1978; Kager et al., 2000; Schiff, 2011; Ullah et al., 2015). Mean field models and phenomenological models developed to explain large-scale brain activity [synchronized activity in a cortical column (Jansen and Rit, 1995)] have also been used to propose mechanisms underlying the generation of slow oscillations. These include (i) a model of thalamocortical interactions, which can generate slow waves important for sleep rhythms (Wilson et al., 2006); (ii) or considering slow regulatory mechanisms with mean field models that induce DC shifts in simulated brain activity (Liley and Walsh, 2013; Jirsa et al., 2014; Lundstrom, 2015; Jafarian et al., 2019a,b; Stefanovski et al., 2019).

The aim of this study is to introduce neither a fundamentally different way to infer a mechanism underlying slow oscillation generation (or its relations to fast oscillation) nor a new mathematical model (or estimation technique) that can emulate transitions into and out seizures with DC shifts. Instead, we aim to illustrate the importance of wide-bandwidth data for capturing the evolution of biological parameters that model critical transitions in brain dynamics. For this, we use a data set obtained from a mouse model of chemoconvulsant-induced seizures using state-of-the-art flexible graphene transistor arrays (Bonaccini Calia et al., 2021). This recording device captures ultraslow potential shifts to high-frequency oscillations from awake brain (Bonaccini Calia et al., 2021), free from movement artifacts. This makes it possible to conclude that recorded ultraslow potential shifts and oscillations result from neuronal dynamics. For the experiments in this study, we choose to model two electrographic traces that captured the transition to seizures either with or without a prominent pre-seizure DC shift. We also high-pass-filtered these data (at 0.3Hz) to emulate recordings that are conventionally available in clinical practices. We infer neural dynamics from these data sets using nonlinear unscented Kalman filter method (Sitz et al., 2002; Voss et al., 2004; Sarkka, 2007). We finally compare the ensuing estimate of synaptic physiology from each data sets and show that for the data where the DC shift is small, or removed with high-pass-filtering, the outcome of estimations is well correlated, whereas in data with a significant infraslow pre-seizure component, the estimated synaptic physiology is considerably different. These simulations in turn suggest that wide-bandwidth recordings are best suited to track changes in synaptic parameters from data that capture paroxysmal transitions.

In summary, in this study, we establish novel simulation platforms to demonstrate the necessity and importance of wide-bandwidth electrophysiological recordings (DC shifts and infraslow activity to fast oscillation) for tracking the evolution of synaptic physiology in a biologically informed model. We use an unscented Kalman filter method to track the dynamics of key synaptic physiology in a mesoscale model of brain activity using real animal brain activity (*in vivo*) with paroxysmal transitions that may or may not be accompanied with DC shifts. We show that high-pass-filtered versions of these brain recordings (which are conventionally available to clinicians) are not reliable for inferring biological parameters. Therefore, we demonstrate the necessity of DC shifts and infraslow activity in electrophysiological data for inferring intrinsic mechanisms related to the transition to seizure.

## Materials and Methods

### Chemoconvulsant Animal Model of Seizures

Animal experiments in this study were conducted in accordance with the U.K. Animal (Scientific Procedures) Act 1986, with approval from Home Office (license PPL70-13691) and the local ethics committee at the Institute of Neurology, University College London. The data were recorded in a chemoconvulsant animal model of seizures (using 4-aminopyridine drug) from awake, unanaesthetised mice. Recently developed state-of-the-art graphene-based transistor arrays were implemented for wide-bandwidth electroencephalography recordings from the surface of cortex (epicortical grids of 4 × 4 which are placed over the somatosensory cortex), as well as laminar recordings through a cortical column, in the visual cortex (intracortical depth electrode with 14 recording sites) (see Bonaccini Calia et al. (2021) for further details).

**Figure 1:**
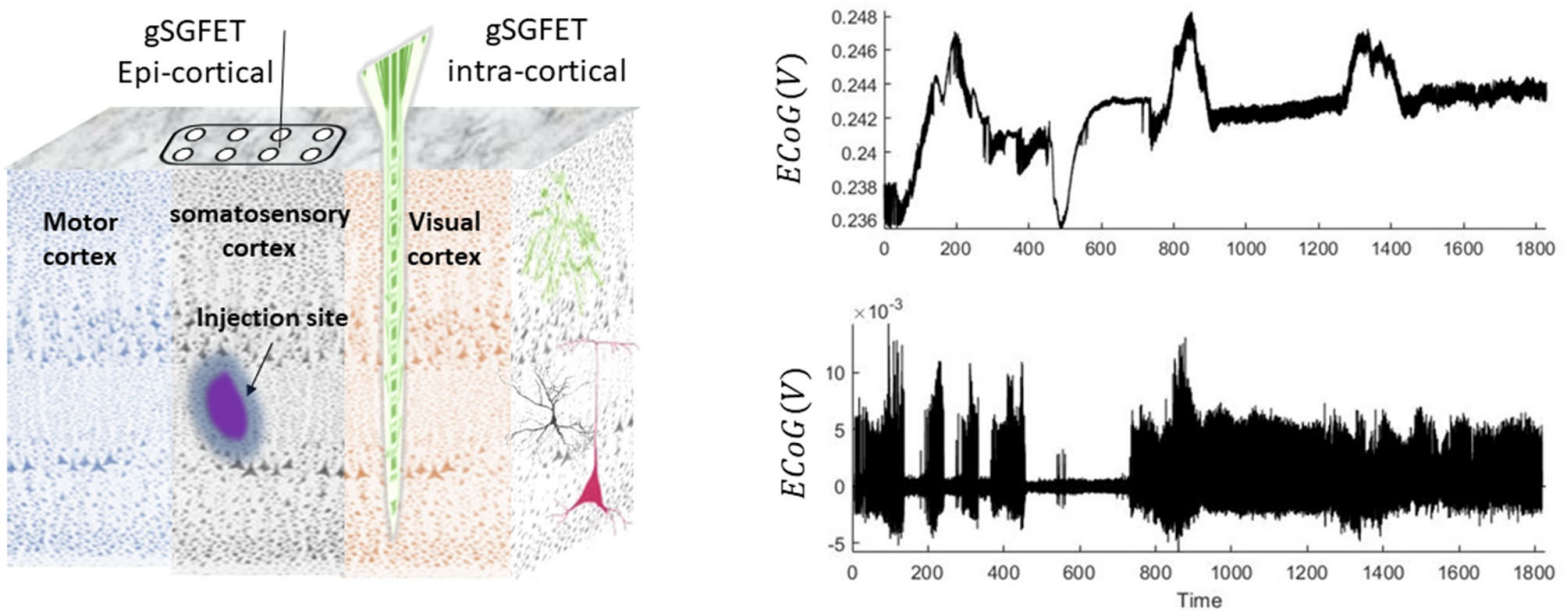
Schematic and examples of the animal recordings. In this experiment, seizures were induced by injection of 4-aminopyridine. Epicortical (using a grid of 4 × 4) and intracortical (14 laminar locations) neuronal recordings are performed using graphene solution-gated field-effect transistors (gSGFET) from the somatosensory cortex (SC) and visual cortex (VC), respectively. The right panel shows a 30-min trace of wideband intracortical (top) and its high-pass-filtered (0.3 Hz) (bottom plot) data that were recorded from the superficial layers of the cortex.

Animals were group-housed (to acclimatize for at least 1 week before surgery) on a 12-h/12-h dark/light cycle, where food and water were given *ad libitum*. A surgery was performed to implant a head bar on the mouse skull to allow stable attachment of the animal to a Neurotar chamber for reliable (and effectively movement artifact free) data recordings both from epidural and intracortical columns with a sampling frequency of 9.6 kHz (Bonaccini Calia et al., 2021). Focal injection of 4-aminopyridine (4-AP) (50 mM; 350 nL into the somatosensory cortex region) was performed at a depth of ~500 μm into the cortex using a 33-gauge needle (see Figure 1).

The epi-/intracortical electrodes capture the neuronal activity during the entire experiment. The spread of drug is locally restricted to the site of injection (Rossi et al., 2017). For the aim of this study, we use data from the superficial layers of the cortex located close to the injection site. The trace of the wide-bandwidth and high-pass-filtered data is shown in Figure 1. An interesting feature in these data is that some seizures are accompanied with DC shifts and some without DC shifts. Therefore, a key hypothesis for the data in this study is that there could be differences between biological generators of seizures that are accompanied with or without DC shifts.

### Neural Mass Model

A neural mass model (NMM) describes the electrical activity of a cortical column (which is captured using electroencephalography techniques) through a low-dimensional biological informed dynamical systems (Wilson and Cowan, 1972; Jansen and Rit, 1995). Assumptions that contribute to driving NMM formulation are as follows: (i) synchronized firing of neurons in a cortical column induce observable electrical activity (Mountcastle, 1957; Hubel and Wiesel, 1963; Felleman and Van Essen, 1991), (ii) neurons within cortical columns can be clustered into few populations (because at each layer of the cortex, effectively one type of neurons resides), mean activity of each of which could be modeled independently, and (iii) the mean activity of different populations interacts and shape the mesoscale electroencephalogram recordings (this assumption is supported by the statistical mean field theory) (Cowan, 1969; Deco et al., 2008; Faugeras et al., 2009).

NMMs are minimally biologically informed dynamical systems that approximate interactions between (collective) activities of neurons in a cortical column with few neuronal populations. NMMs are low-dimensional, with few biological parameters (compared to networks of interconnected neurons) and can well recapitulate electrophysiological data (Jansen and Rit, 1995). Crucially, there are formal mathematical links between an interconnected network of single neurons and an NMM (Deco et al., 2008; Faugeras et al., 2009; Veltz and Faugeras, 2010; Faugeras and Inglis, 2015). Collective dynamics of neurons are well approximated by NMMs, while they retain biological realism. The low-dimensional representation of NMMs is well suited for either designing biologically motivated control systems to suppress seizures (Schiff, 2011) or understanding interactions between different brain regions in cognitive tasks (Friston et al., 2011, 2019; Schwartenbeck and Friston, 2016; Shaw et al., 2017; Jafarian et al., 2019c). Therefore, NMMs are suitable for a variety of translational neuroscience applications and can be implemented to study biologically motivated hypothesis regarding underlying generators of brain activity.

One could show (both theoretically and experimentally) that dynamics of a population of many neurons can be governed (or summarized) by static conversion of input mean synaptic activity to firing rates (average of action potentials) and firing rates to mean synaptic activity (scaled by anatomical connection strengths between populations or cortical layers), which is input to other populations (Wilson and Cowan, 1972; Freeman, 1975; Jansen and Rit, 1995).

Mathematically, synaptic potential (*V*) into firing rate conversion is modeled by a sigmoid transformation (σ(.)) as previously described (Wilson and Cowan, 1972).


(1) $$\begin{array}{r} \sigma \left(V,\;V_{th}\right)\;=\frac{e_{0}}{1+\exp\left(-\rho \left(\;V-V_{th}\right)\right)} \end{array}$$

In Equation (1), *e*_0_ is the maximum firing rate, ρ is the slope of the transformation, and *V*_*th*_ is the firing threshold (when input potentials reach half maximum firing rates).

The conversion of the firing rate to the mean postsynaptic membrane potential is modeled by a second-order low-pass filter with an impulse response $h\left(t\right)=A\frac{t}{T}e^{-\frac{t}{\;T}}$ where *t*≥0, *A* is a maximum postsynaptic potential (also known as synaptic gain), and *T* is a synaptic time constant (Freeman, 1975). Postsynaptic potential *V* that is generated by firing rate σ(.) can be calculated simply by convolving the input firing with synaptic kernels, *V*(*t*) = *h* ⊗ σ (the symbol ⊗ represent the convolution), which is equivalent to the following second-order differential Equation (note the second-order differential equation can be equivalently written in terms of two first-order systems)


(2) $$\begin{array}{r} {\left(1+\frac{1}{T}\frac{d}{dt}\right)}^{2}V\left(t\right)\;=\;A\;\sigma \left(t\right) \end{array}$$

**Figure 2:**
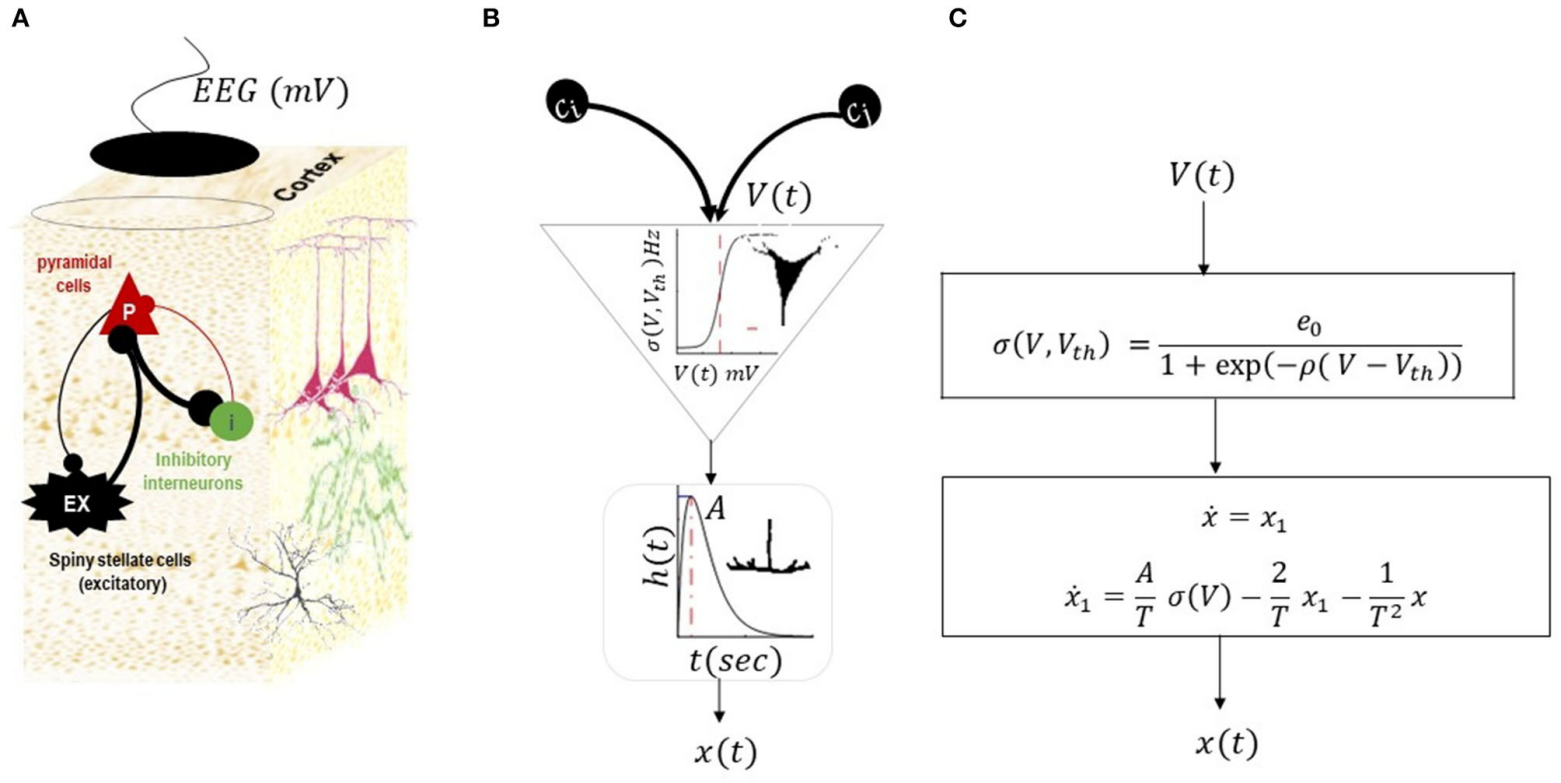
Neural mass model by Jansen and Rit (1995). This NMM has three populations of inhibitory, excitatory, and pyramidal cells, as shown in **(A)**. The intrinsic connections between populations can be excitatory (black lines) or inhibitory (red lines). The membrane potential of pyramidal cells is considered as simulated EEG data. **(B)** Illustrates a conversion operator within a population. Each population in the NMM receives synaptic inputs from other populations (scaled by inter-regional connections (*C*_._)). The inputs are then converted to the mean firing rate using sigmoid transformations (average of action potential of many neurons with thresholds *V*_*th*_). The ensuing firing rate is then converted into postsynaptic potentials using second-order low-pass with kernel *h* (*t*) = *tAT*^(−1)^ exp (*tT*^(−1)^) (where *A* is the maximum postsynaptic potential and *T* is the synaptic time constant). The postsynaptic potential is then scaled by intrinsic connectivity and drives other populations. The list of parameters in the model and their expected range are provided in Table 1. The equivalent mathematical equations is shown in **(C)**.

The input–output relations that govern the dynamics of a neuronal population are largely similar among different NMMs, although the number of neural populations and their connections (scale factor to synaptic potentials) to each other can be varied between NMMs. In this study, we use an NMM that was developed by Jansen and Rit (1995) and is shown in Figure 2.

This NMM explains the activity of a cortical column through interactions of three populations, namely, (i) excitatory spiny stellate cells (denoted by *ex* in model equations) situated in layer 4 of a typical cortical column, and they receive endogenous input from other regions; (ii) inhibitory cells (denoted by *i* in model equations) that are distributed across the columnar organization; and (iii) pyramidal cells (denoted by *p* in model equations) whose activity is predominantly captured by recording electrodes. The equation of the NMM associated with the structure in Figure 2 can be written as follows:


(3) $$\begin{array}{r} \begin{array}{c} \overset{∙}{x_{p}}=x_{p^{*}}\\ {ẋ}_{p^{*}}=\frac{A_{p}}{T_{p}}\;\sigma \left(x_{ex}-x_{i}\right)-\frac{2}{T_{p}}\;x_{p^{*}}-\frac{1}{T_{p}^{2}}x_{p}\\ {ẋ}_{ex}\left(t\right)=x_{ex^{*}}\\ {ẋ}_{ex^{*}}=\frac{A_{ex}}{T_{ex}}\left(\omega +\left(0.8\;\times C\right)\times \sigma \left(C\times x_{p}\right)\right)-\frac{2}{T_{ex}}\;x_{ex^{*}}-\frac{1}{T_{ex}^{2}}x_{ex}\\ {ẋ}_{i}\left(t\right)=\;x_{i^{*}}\\ {ẋ}_{i^{*}}\left(t\right)=\frac{A_{i}}{T_{i}}\left(\left(0.25\;C\right)\times \sigma \left(0.25\times C\times x_{p}\right)\right)-\frac{2}{T_{i}}\;x_{i^{*}}-\frac{1}{T_{i}^{2}}x_{i} \end{array} \end{array}$$

In Equation (3), the vector $\left[x_{p},\;x_{p^{*}},\;x_{ex},\;x_{ex^{*}},x_{i},\;x_{i^{*}}\right]\in \;R^{6}$ represents the physiological states, either the synaptic activity of the populations (*x*_*p*_, *x*_*ex*_, *x*_*i*_) or their first-order derivative ($x_{p^{*}},x_{e^{*}},x_{i^{*}}$). The fixed parameters in Equation (3) are synaptic parameters for each population $\left(A_{.},\frac{1}{T_{.}}\right)$, inter-region connections which are defined by scaling the universal constant *C*, and parameters of sigmoid transformations. The endogenous input to the model is ω, which can be a random, constant, or smooth function (see Table 1 for the range of variations for NMM parameters). The membrane potential of pyramidal cells is considered simulated electroencephalogram data. Hereinafter, we rewrite the NMM equation in the form of a general canonical dynamical systems as follows:


(4) $$\begin{array}{r} \begin{array}{c} ẋ\left(t\right)=f\left(x,\theta \right)+n\left(t\right)\\ \overset{∙}{\theta }\;=0 \end{array} \end{array}$$

In Equation (4), $x=\;\left[x_{p},\;x_{p^{*}},\;x_{ex},\;x_{ex^{*}},x_{i},\;x_{i^{*}}\right]\in \;R^{6}$ and represents the biological states, the right hand side of equation 3 is denoted by a nonlinear function $f\left(.\right)\in \;R^{6}$, the vector *n*(*t*) is the endogenous fluctuations (which can be modeled as a constant number, smooth function, or noise), and θ is the set of constant parameters in the model (thereby their derivative is zero as written in the second line of the Equation (4).

**Table 1:** Parameters of the NMM and their variation range.

**Acronyms**	**Description**
A_*p*_,ex	Maximum PSP of pyramidal and excitatory population [2.5–10] mV
A_*i*_	Maximum PSP of inhibitory population [3–100] mV
T_*i*_	Synaptic Time constant of inhibitory population 1/50 (s)^−1^
T_*p*_,ex	Synaptic Time constant of pyramidal and excitatory population 1/100 (s)-1
C	Connectivity constant [60–1,350]
*V*_*th*_	Firing threshold [2–7] mV
ρ	Slope of sigmoid function 0.56 (s)^−1^
*e*_0_	Maximum firing rate 5 (s)-1
ω	Endogenous random Gaussian fluctuation with mean μ [160–260] and variance Σ = 1.

The most obvious way to explore the origin of the oscillations in the brain using the NMM is forward simulation of different sets of parameters and initial conditions. For instance, in Jansen and Rit (1995), the model was simulated for different values of connections between populations (the universal connection constant *C* was varied from 68 to 675) and brain rhythms, such alpha and spike wave discharges, were replicated. The NMM is used to explore a path between normal and pathological activity in parameter space by changing the balance between maximum postsynaptic responses of excitatory and inhibitory connections (e.g., Wendling et al., 2002, 2005). Motivated by these studies and electrophysiological knowledge about the animal model of seizures in this study, we assume that an underlying cause for the transitions can be explained by changes of postsynaptic potentials of inhibitory populations (*A*_*i*_).

Sophisticated mathematical analysis such as bifurcation can be performed to formally study the behavior of the NMM as some parameters (one or two) are changed (e.g., Grimbert and Faugeras, 2006; Spiegler et al., 2010; Touboul et al., 2011). Despite valuable information informed from bifurcation analysis, practically, it can only be applied to one of two parameters. In addition, this form of analysis can only be applied to a deterministic NMM (i.e., the input to the model is a constant). In other words, the interpretation of the bifurcation structure of random bifurcation is still in its infancy (e.g., Crauel and Flandoli, 1994, 1998; Callaway et al., 2017). However, it is possible to prove that the solution of the NMM is stationary for a constant set of parameters, with stationary noise as its input (Faugeras et al., 2009; Veltz and Faugeras, 2010, 2011; Faugeras and Inglis, 2015). In this study, we re-confirm these findings by simulating the global invariant measures of the NMM for different parameters to illustrate that only one type of activity can be viewed using an NMM with fixed parameters.

In this study, first, we estimate constant parameters of the model based on spectral features in a stationary segment of data using dynamic causal modeling (also known as variational Bayesian inversion of a nonlinear system under Laplace assumption) of spectral response (Friston et al., 2012; Jafarian et al., 2021). The assumption in dynamic causal modeling of cross-spectral response is that neural dynamics rest at a stable equilibrium and oscillations (finite deviation from baseline equilibrium) are induced due to random exogenous input (Lopes Da Silva et al., 1974; Friston et al., 2012). [See Friston et al. (2012) for details of procedures].

We used the ensuing identified model and tracked changes of postsynaptic potentials of the inhibitory population in an NMM using a continuous–discrete unscented Kalman filter (UKF) (Voss et al., 2004; Jafarian et al., 2019b) from data that exhibit transitions into and out of seizures. The UKF is a class derivative-free stochastic filter that can recursively estimate hidden states of partially observed nonlinear dynamical systems from real data. The UKF approximates posterior estimates of states using Gaussian distribution, which in turn makes this filter computationally efficient while accurate (Sitz et al., 2002; Voss et al., 2004; Sarkka, 2007).

The generative model for tracking the inhibitory gain in the NMM for a given data using the unscented Kalman filter (UKF) is as follows:


(5) $$\begin{array}{r} \begin{array}{c} ẋ\left(t\right)=f\left(x,\theta \right)+n\left(t\right)\\ \overset{∙}{\theta }\left(t\right)=0+n_{{}^{*}}\left(t\right)\\ y_{n}\;=\;H\;{\left[x\left(t_{n}\right),\;\theta \;\left(t_{n}\right)\right]}^{{}^{\prime }}+e \end{array} \end{array}$$

The first line of Equation (5) is similar to the general equation of the NMM. The second line expresses the dynamics of inhibitory gains, which is recovered from data using the UKF method. We consider additive uncertainty (with very small variations $n_{{}^{*}}\;\sim N\left({0,10}^{-8}\right)$ to the equation of motions of θ(= *A*_*i*_) to allow the UKF algorithm to adjust the values of the synaptic gains from the data (Schiff, 2011; Jafarian et al., 2019a). It should be noted that in the context of state estimations from data using any form of Kalman filter, the role of noise in hidden states is interpreted as uncertainty (Sitz et al., 2002; Voss et al., 2004). The left hand side of the third line of Equation (5) is discrete real data (sampled recordings), and the right hand side expresses how the solution of the generative model is linked to observational data (here, the membrane potential of pyramidal cells which is defined by the vector *H* = [0 0 1 0 −1 0 0]'), and *e* is the random effect which has a normal distribution.

## Results

### Forward Simulation of NMM

**Figure 3:**
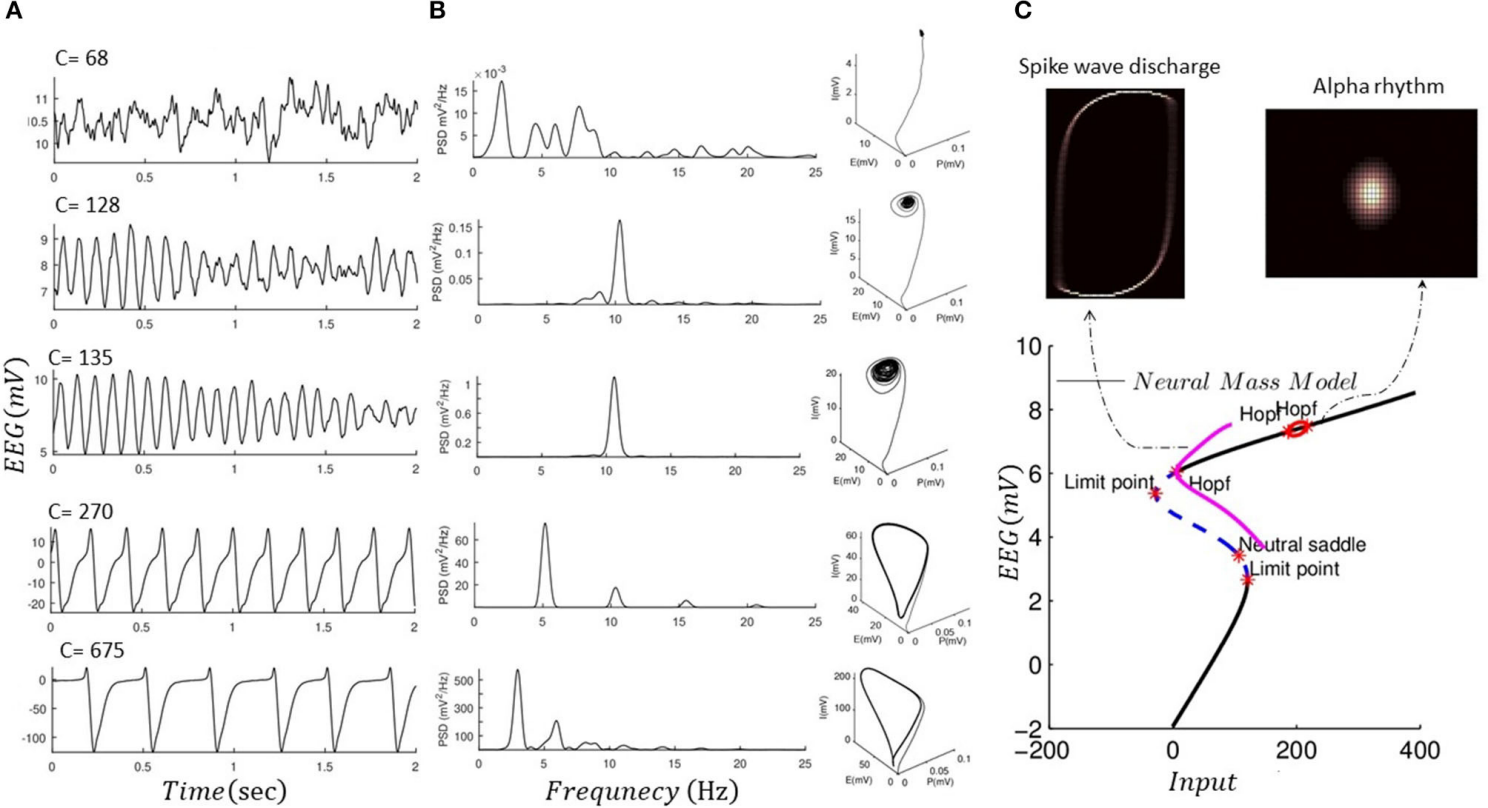
Forward simulation of NMM. **(A)** Forward simulation of the noise-driven NMM for different connectivity constants as shown in each plot (fixed parameters among these simulations are given in Table 2). **(B)** Illustrates the power spectral response (PSD) of the simulated EEG data using the NMM in panel I, as well as three-dimensional phase space of the model based on finite time simulation (P, I, and E denote activity of pyramidal, inhibitory, and excitatory cells). **(C)** Shows a projected bifurcation plot (i.e., with respect to the output of the model) when input to the model is changed (connectivity constant 120). In addition, we plot the invariant measure of the model (projected into two dimensions of the excitatory and inhibitory cells) which illustrates that the solution of the NMM would not exhibit transitions. The bifurcation diagram was produced using MatCont software (KuznetSOv et al., 2019), and invariant measures were produced using GAIO code package (Fiedler, 2012).

We performed forward simulation of a noise-driven NMM akin to Jansen and Rit (1995) for different values for intrinsic connections (*C* = 68, 128, 135, 270, 675) and replicated alpha rhythms and spike wave discharge, as shown in Figure 3. NMM is simulated using a stochastic RK method as explained in Wilkie (2004). In Figure 3, we also show the global attractor of the NMM for alpha rhythm and spike wave activity. The shape of the global attractor illustrates the region in the model phase space that is almost certainly occupied by the noise-driven model in the limit. Because the phase space that is occupied by the global attractor is similar to its finite time simulations, one could assume that the noise-driven model for different fixed parameters could generate alpha and epileptic discharge without any transitions. Finding the global attractor can be seen as a complementary analysis to recent mathematical proof that the noise-driven NMM could generate stationary solutions with fixed parameters (Faugeras et al., 2009; Veltz and Faugeras, 2010, 2011). Here, we also provide a local bifurcation plot of the NMM, similar to Grimbert and Faugeras (2006), which can be used to study the system equilibrium properties as its input is altered. As mentioned, the conventional bifurcation analysis can be applied to deterministic systems and could reveal information relevant to the underlying mathematical features that support different sorts of activities. For instance, in the bifurcation plot in Figure 3, one could argue that limit cycle activity may be associated with epileptic activity.

**Figure 4:**
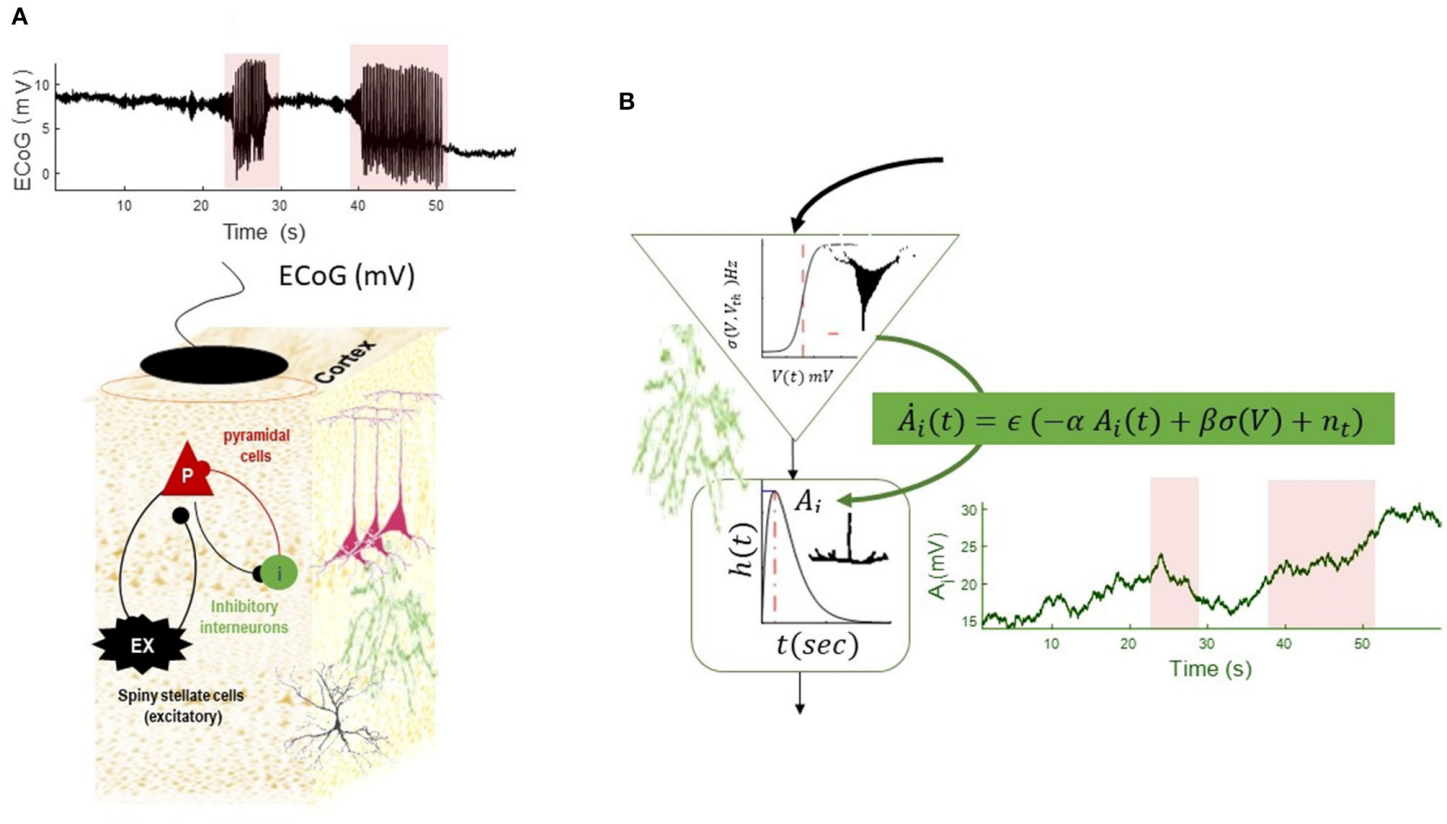
Simulation of a slow–fast neural mass model **(A)**. The equation of the NMM is augmented with slow dynamics for the synaptic gain of the inhibitory population **(B)**. The dynamics of inhibitory gain resemble changes of synaptic plasticity and are modeled by Ȧ_*i*_ = ϵ (−α*A*_*i*_+βσ (*V*)+*n*(*t*)), where the recovery time constant of the gain is denoted by α(Hz) and fast- to slow-state constant is denoted by β, noise at the slow scale is denoted by *n*(*t*), and time-scale separation parameter is ϵ. (In the absence of any firing rate input, the synaptic gain converges to a constant value with the rate of α). The slow equation of motion in this simulation is ${Ȧ}_{i}=0.1\;\left(-0.06A_{i}+5\;\sigma \;\left(V\right)+n\left(t\right)\right)\;\left(n\sim N\left({0,10}^{-6}\right)\right)$. The simulated EEG trace shows transitions into and out of seizures, as well as DC shifts as the result of increasing synaptic plasticity at the seizure onset, which is decreasing toward the end of seizures. The termination of the second seizures displays post-ictal silencing and has less variance than pre-seizure activity. This phenomenon is conventionally understood as a lack of energy in neuronal activity. The fixed parameters of this simulation are given in Table 2.

To simulate transition into and out of seizures, we equipped the NMM with slow dynamics of postsynaptic gain of inhibitory populations, as shown in Figure 4. In this simulation, the inhibitory gain is regulated by input firing rates and also has a period of recovery time. This effectivity resembles alteration of synapse gain and plasticity (e.g., Jafarian et al., 2021). As can be seen in Figure 4, this slow–fast NMM can show transitions to and from seizures, as well as alterations in DC shifts as the plasticity of inhibitory populations is altered.

### Tracking Synaptic Physiology Using Wide-Bandwidth and High-Pass-Filtered Data

We infer baseline parameters (noise inputs and synaptic gains), while others are fixed (see Table 2 for details) from a stationary segment of real data using dynamic causal modeling of cross-spectral density (Friston et al., 2012). We assume that all ensuing baseline parameters remain unchanged during transitions to seizures in data, except the synaptic gain of the inhibitory population. This effectively models pathological alterations of excitatory-inhibitory balance during paroxysmal transitions (Wendling et al., 2002).

**Table 2:** Fixed parameters of the NMM in different simulations of this study.

**Parameter**	**Figure 3**	**Figure 4**	**Figures 5, 6**
*A*_*p*_(*mV*)	3.25	3.25	3.147
*A*_*ex*_(*mV*)	3.25	3.25	2.831
*A*_*i*_(*mV*)	22	22	26.072
*T*_*i*_(*s*)	50	50	50
*T*_*p*_(*s*)	100	110	100
*T*_*ex*_(*s*)	100	110	100
*V*_*th*_(*mV*)	6	6	5
*C*	[68–675]	145	190
ρ(*Hz*)	0.56	0.56	0.56
*e*_0_(*Hz*)	5	5	5
ω ~ ℵ(μ, Σ)(*Hz*)	ℵ(220, 1.5)	ℵ(160, 1.5)	ℵ(200, 1.2)

**Figure 5:**
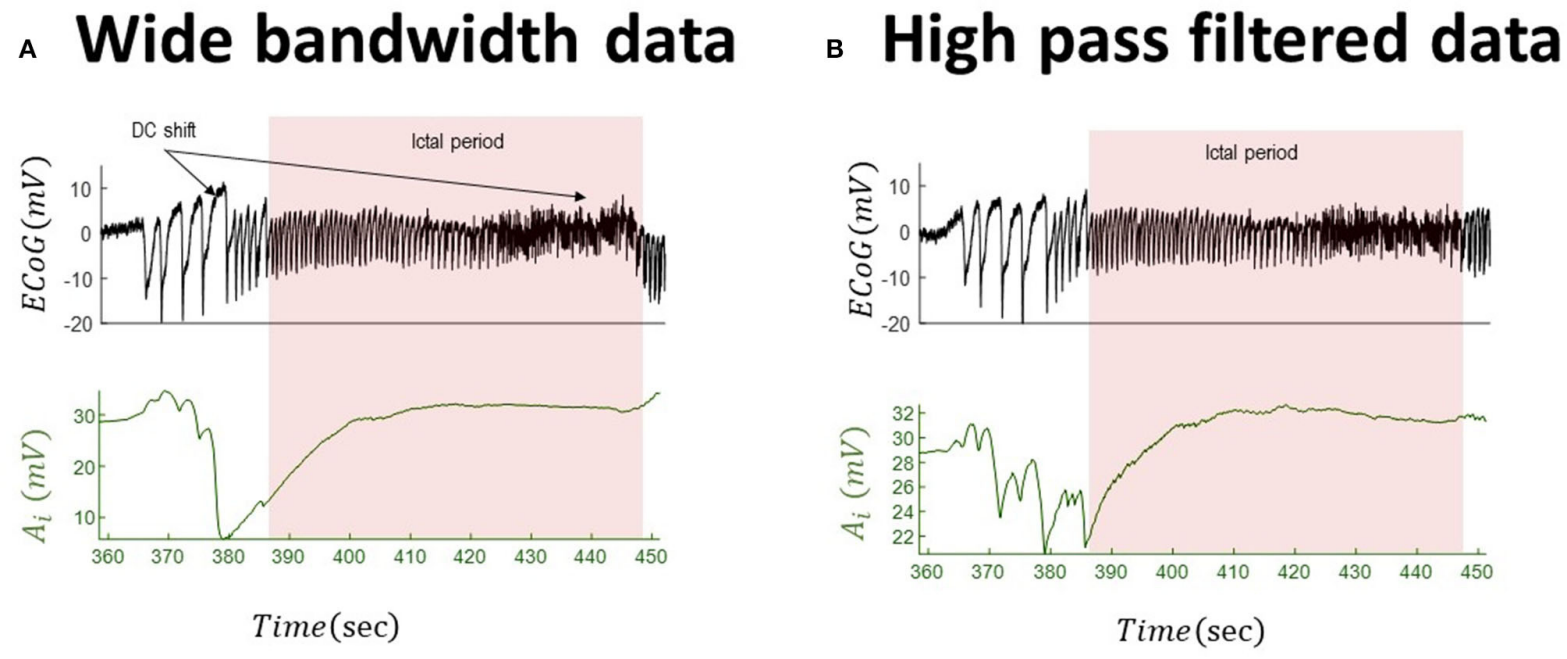
Tracking seizures dynamics from wide-bandwidth (effectively without DC shifts) and high-pass-filtered data with paroxysmal transition. The left panel **(A)** shows the wide-bandwidth data at the top and inferred synaptic gains of inhibitory population at the bottom using the UKF approach (the uncertainty around the inhibitory gain in Equation (5) is *n*~*N*(0, 10^−8^) in this simulation). The synaptic gain tracks changes of the slow wave discharges and increases during the seizures. The right panel **(B)** shows the high-pass-filtered version of the wide-bandwidth data and the inferred trajectory of inhibitory gains. Comparing the behavior of the inferred synaptic gains suggests a similar behavior, which is due to the fact the DC shift in the wide-bandwidth data set is small. The fixed parameters of the NMM used in this simulation are given in Table 2. These data are a part of the recording trace in Figure 1.

We employed an unscented Kalman filter to track changes of inhibitory gain in two sets of real data (scaled by a factor of 10^4^ to make its variance in the range of the NMM output) and their high-pass-filtered version (which mimics data that are usually available from conventional electrographic devices). We start by tracking the inhibitory gains for the full-bandwidth data with a small DC shift. This effectivity implies that the high-pass-filtered version and original wide-bandwidth data are very similar. The outcome of tracking the dynamics of inhibitory gain is shown in Figure 5 for both wide-bandwidth and high-pass-filtered data. The inferred trajectories of inhibitory gain have very similar behavior. In this case, the inferred inhibitory gain is altered as the large spikes appear in the data and increase during the recurrence of seizures.

**Figure 6:**
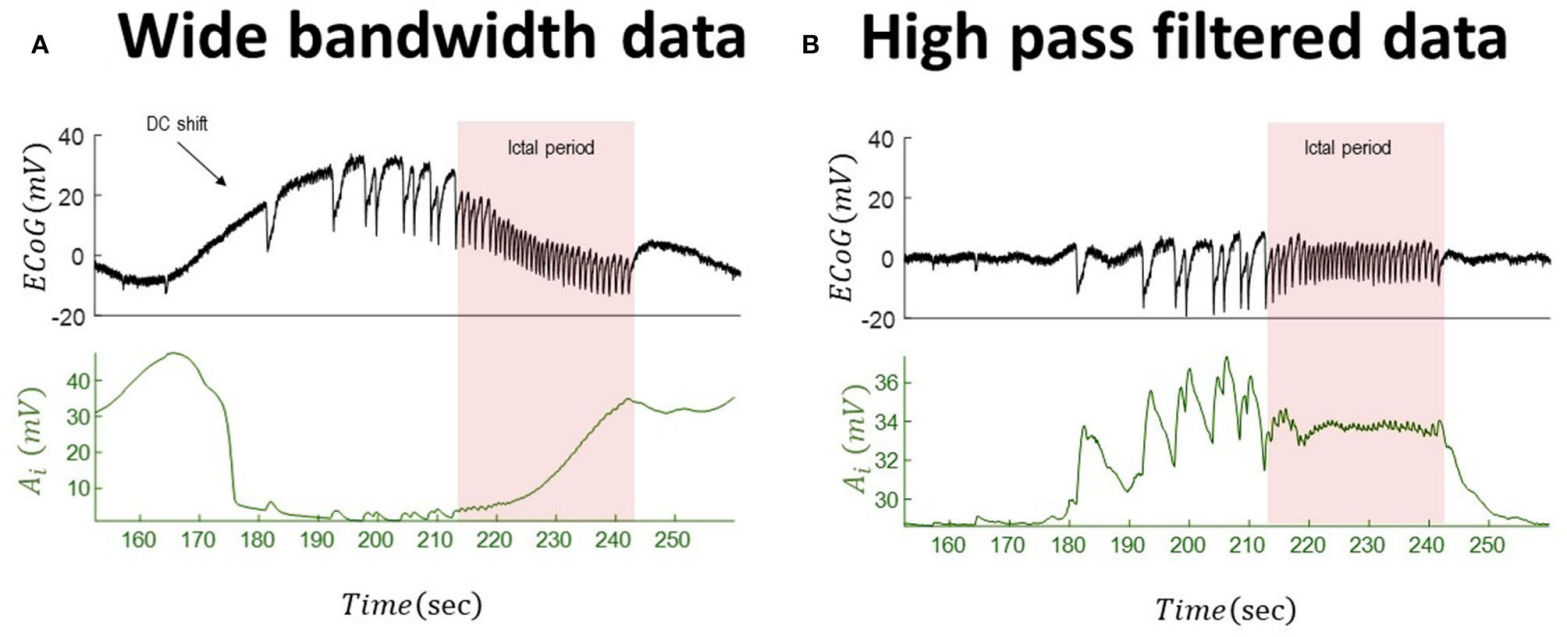
Tracking seizure dynamics from wide-bandwidth data (with a DC shift) and its high-pass-filtered with paroxysmal transitions. The left panel **(A)** shows the wide-bandwidth data at the top and inferred synaptic gains of inhibitory population at the bottom using the UKF algorithm (the uncertainty around the inhibitory gain in Equation (5) is *n*~*N*(0, 10^−8^) in this simulation). The synaptic gain tracks changes of the infraslow activity and increases during the seizures. The right panel **(B)** shows the high-pass-filtered version of the data and the inferred trajectory of inhibitory gains. The behavior of the inferred synaptic gains suggests an increase during the paroxysmal transitions and return to baseline after seizures. Comparing the results suggests that infraslow changes potentially play a key role for understanding underlying generators of the data. In fact, the inferred synaptic gains from the wide-bandwidth data show a totally different behavior from that of high-pass-filtered data. The fixed parameters of the NMM used in this simulation is given in Table 2. These data are a part of the recording trace in Figure 1.

We repeat the analysis for the wide-bandwidth data with significant DC shifts and for the same-signal subsequently high-pass-filtered data. The inferred inhibitory gains for both wide-bandwidth and high-pass-filtered data are shown in Figure 6. In this simulation, there are significant differences between behavior (effectively in the opposite direction) of the recovered slow evolution of inhibitory gains from both data. In the high-pass-filtered version of the data, the synaptic gain increases before paroxysmal transitions and returns to its baseline after seizures. The recovered trajectory of the inhibitory gains from wide-bandwidth data shows a totally different behavior as it starts to decrease during the pathological activity and then increases during seizures.

These four examples may be seen as a proof of concept that all information in the recorded data needs to be considered for capturing the underlying dynamics of biological generators of data, particularly when modeling data with paroxysmal transitions into and out of seizures, which requires information about all frequency ranges from slow to very high.

## Discussion

In this study, we illustrate the importance of wide-bandwidth data for translational neuroscience applications, in particular for modeling data with paroxysmal transitions into and out of seizures. The importance of ultraslow potential shifts and oscillations has been known for many years, and their potential clinical usage, for example, improving localization of the epileptogenic zone, has been proven. Unfortunately, due to the poor performance of standard electrodes used clinically to record these slow brain signals accurately and with high fidelity, they are seldom recorded, reported, or studied. Here, we discuss a potential advantage of wide-bandwidth DC-coupled data over conventional recording settings for inferring parameters in a biological model through simple but intuitive examples. We show that the DC shift in data provides a different estimation of the synaptic physiology from that of high-pass-filtered data and thus lacking ultraslow information. As shown in this article, the estimation from high-pass-filtered and wide-bandwidth DC-coupled data may provide a totally different picture regarding underlying generators of data.

There are limitations to modeling the study presented in this article, and therefore, our findings should be considered only as proof of concept to motivate usage and uptake of wide-bandwidth DC-coupled data for translational neuroscience applications. We assume only one parameter in the NMM is responsible for transitions into and out of seizures (despite this, the modeling approach can replicate the pathological imbalance between excitation and inhibition). The excitation–inhibition balance is a generic hypothesis that has been be studied as a local network of interconnected neurons, as well as mesoscale or macroscale models under the same principals to explain brain activity in health and disease. Alteration of the excitation–inhibition balance in the NMM (Wendling et al., 2002) is an intuitive approach to explain seizure initiation and termination. The goodness of this hypothesis can be assessed and refined (by selecting different sets of connections in the model) through predictive validity tests (i.e., whether the prediction of the model is similar to observations made from animal data). There may be different biological contributors to the initiation and termination of seizures (Kramer et al., 2012; De Curtis and Avoli, 2015), which need to be considered. The key message of this article is that wideband data are required for modeling the underlying causes of paroxysmal transitions, irrespective of parameter selection. It should be noted that we consider only one parameter to explain transitions into and out of seizures. Having said that, application of the UKF (or any forms of Bayesian filtering) for tracking of more than one parameter could be ill-posed due to symmetry problems. This refers to compensation of evolution of one parameter by other parameters in statistical inference of parameters in partially observed dynamical systems (Haykin, 2004; Simon, 2006). In the animal model used in this study, we assume that seizures initially arise focally in the region injected by chemoconvulsant. Therefore, we only investigate changes of parameters associated with the first induced seizures and from one region of the brain. Having intra- and epicortical data using our novel recording technology, we expect spatial–temporal neuronal modeling can unpack more about the relation between ultraslow potential shifts, infraslow oscillations, and paroxysmal transitions.

As future research, one could employ the bifurcation theory to explore regions in the parameter space in which seizures are accompanied with/without DC shifts (e.g., similar to Saggio et al., 2020), although this information can only be investigated for a limited number of parameters. The ensuing finding can potentially be useful for model inversion (for instance, in data with paroxysmal transitions without DC shifts, one could restrict the parameter search space to those in which seizures are not accompanied with slow potential changes). One could also consider slow evolution for parameters and investigate the onset/offset of seizures with infraslow oscillations. Potentially, one could apply a similar approach to this study and investigate underlying bio-generators of the data (e.g., Jafarian et al. (2019a), Jafarian et al. (2019b)). Based on the assumption that the recording contact is in the seizure onset zone, rather than an area of propagation, several points can be made. It may be possible to conceptualize differences between underlying generators of these seizures where the insights into evolution of biological parameters (i.e., extracellular potassium buffering vs. decrease in inhibition) can play a key role for designing treatment. Clinicians may use insights from modeling patient-specific seizures and then apply different interventions (classes of drugs or neuromodulatory stimulations) (Schiff, 2011)] to optimize the therapeutic efficacy of seizure suppression. In theory, we could specify/assign slow dynamics to different parameters in the neuronal model, perform model inversion, and by using Bayesian model comparison explore the likely model of seizures with and without DC shifts. The likely model can be experimentally tested by assessing specific ways to control animal seizures and refined based on the outcome of the perturbations (Schiff, 2011).

## Data Availability Statement

The data in this paper may be available under reasonable request to authors.

## Ethics Statement

Animal experiments in this paper were conducted in accordance with the United Kingdom Animal (Scientific Procedures) Act 1986, with approval from Home Office (license PPL70-13691) and the Local Ethics Committee at the Institute of Neurology, University College London.

## Author Contributions

AJ: conceptualization, methodology, software, validation, formal analysis, writing—original draft, data curation, and visualization. RW: conceptualization, methodology, data acquisition and curation, and review and editing. Both authors contributed to the article and approved the submitted version.

## Funding

RW is funded by a Senior Research Fellowship awarded by the Worshipful Company of Pewterers. This work has received funding from the European Union's Horizon 2020 research and innovation programme under Grant Agreement No 881603 (GrapheneCore3).

## Conflict of Interest

The authors declare that the research was conducted in the absence of any commercial or financial relationships that could be construed as a potential conflict of interest.

## Publisher's Note

All claims expressed in this article are solely those of the authors and do not necessarily represent those of their affiliated organizations, or those of the publisher, the editors and the reviewers. Any product that may be evaluated in this article, or claim that may be made by its manufacturer, is not guaranteed or endorsed by the publisher.
